# Circular RNAs perspective: exploring the direction of immunotherapy for colorectal cancer

**DOI:** 10.3389/fonc.2025.1554179

**Published:** 2025-04-11

**Authors:** Yanlin Cao, Yuxing He, Lingshan Liao, Lixin Xu

**Affiliations:** ^1^ Department of Pathology, Changde Hospital, Xiangya School of Medicine, Central South University, Changde, Hunan, China; ^2^ The First Clinical Medical College of Guangdong Medical University, Zhanjiang, China; ^3^ Department of Medical Laboratory Medicine, Changde Hospital, Xiangya School of Medicine, Central South University, Changde, Hunan, China; ^4^ Neurosurgery Department, Changde Hospital, Xiangya School of Medicine, Central South University, Changde, Hunan, China

**Keywords:** circular RNA, colorectal cancer, immunotherapy, diagnosis, precision medicine

## Abstract

Circular RNAs (circRNAs) are multifaceted molecules that play a pivotal role in regulating gene expression at both transcriptional and post-transcriptional levels. Their expression is highly tissue-specific and developmentally regulated, making them critical players in various physiological processes and diseases, particularly cancer. In colorectal cancer, circRNAs exhibit significantly dysregulated expression patterns and profoundly influence disease progression through diverse molecular mechanisms. Unraveling the complex roles of circRNAs in modulating colorectal cancer immunotherapy outcomes highlights their potential as both promising biomarkers and therapeutic targets. Moving forward, advancements in circRNA-based therapeutic strategies and delivery systems are poised to transform precision medicine, enabling early colorectal cancer diagnosis and improving patient prognosis.

## 1 Introduction

According to the latest data from the International Agency for Research on Cancer (IARC), approximately 1.926 million new cases of colorectal cancer (CRC) were projected globally in 2022, accounting for about 9.6% of all malignant tumors. CRC-related deaths were estimated at 904,000, representing 9.3% of all cancer-related fatalities (1). CRC ranks as the third most diagnosed cancer worldwide, following lung and breast cancers, and is the second leading cause of cancer-related deaths, surpassed only by lung cancer. In China, the 2022 National Cancer Center report identified CRC as the second most frequently diagnosed cancer and the fourth leading cause of cancer-related mortality (2). Overall, CRC remains a major public health challenge, ranking among the top five cancers in both incidence and mortality globally and in China, and contributing significantly to the global disease burden.

The management of CRC combines surgical and non-surgical therapies. Surgical resection remains the cornerstone and primary curative option. However, approximately 25% of patients present with distant metastases, complicating treatment. Evidence suggests that resecting the primary tumor can improve survival (3, 4). Treatment strategies vary by disease stage. For locally advanced rectal cancer, neoadjuvant radiotherapy enhances complete resection rates and reduces recurrence risk. Patients achieving clinical complete remission (cCR) may opt for a watch-and-wait approach, avoiding surgery (5, 6). Stage III CRC patients benefit from adjuvant chemotherapy to lower recurrence rates. For metastatic CRC, personalized therapy, including RAS and BRAF mutation testing, is standard. Immunotherapy, now the fifth major treatment modality, has shown promising results, particularly in neoadjuvant settings for locally advanced and metastatic CRC (4, 7–9).

Recent studies highlight the unique expression profiles of circRNAs in various cancers, making them promising tools for early tumor detection (10). CircRNAs, a distinct class of non-coding RNAs, form closed-loop structures through back splicing, lacking a 5’ cap and 3’ poly(A) tail. This structure provides exceptional stability and conservation, as they resist exonuclease degradation (10–12). Produced via exon or intron cyclization, circRNAs play critical roles in cancer by acting as miRNA sponges, translation templates, and gene expression regulators (13, 14). In CRC, circRNAs show distinct expression patterns in serum, tissues, and exosomes, significantly influencing disease progression (15). For instance, circPTK2 promotes epithelial-mesenchymal transition (EMT) in CRC by interacting with waveform proteins, serving as a potential biomarker and therapeutic target for metastatic CRC (16). Similarly, circZNF800 enhances tumor stem cell properties and CRC progression, with CRISPR Cas13d-based knockdown showing therapeutic potential (17). Additionally, circRERE-AAV inhibits tumor growth and enhances anti-PD-1 therapy efficacy, highlighting its role in CRC immunotherapy (18). This review explores the emerging roles of circRNAs in CRC, focusing on their diagnostic, prognostic, and therapeutic applications, particularly in immunotherapy. Advancing understanding of circRNA biology may lead to innovative CRC management strategies.

## 2 Biological structure and function of circRNAs

### 2.1 Definition and structure

In 1976, German scientists, including Heinz L. Sanger, discovered circRNA, a ring-shaped RNA molecule with a covalent bond linking its 3’ and 5’ ends, formed via back-splicing or a lasso mechanism. This circular structure enhances stability by resisting ribonuclease degradation, surpassing linear RNAs in durability. Most circRNAs are cytoplasmic, with a smaller fraction localized in the nucleus. They play key roles in neural development, tumorigenesis, immune responses, and gene regulation (19). CircRNAs possess distinctive features (10): (1) Strong stability, (2) Specificity, (3) Enrichment, (4) Evolutionary conservation, (5) Non-cap-dependent translation, and (6) Low immunogenicity. Their low immunogenicity reduces innate immune activation and dendritic cell maturation, preventing immune responses to encoded therapeutic proteins. CircRNAs are synthesized in eukaryotic cells through two pathways: (1) back-splicing, where the downstream splice donor joins the upstream splice acceptor, forming a closed loop, or (2) intronic lasso formation during linear splicing. Research highlights their critical roles in maintaining stem cell pluripotency and directing differentiation, as well as in tissue development, maintenance, and regeneration (20, 21).

### 2.2 Function and regulation

**Figure 1:**
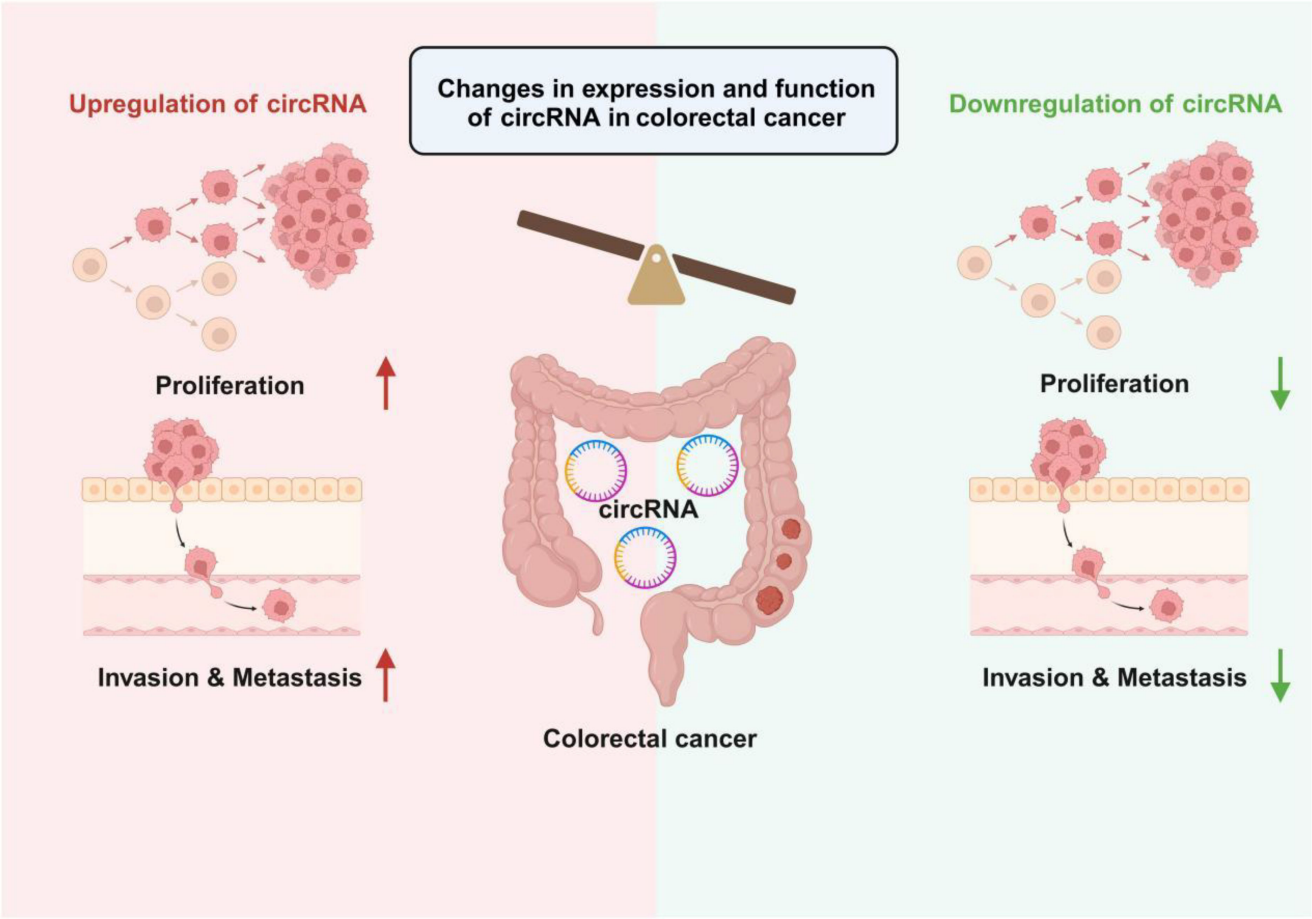
Changes in circRNA expression levels in colorectal cancer and their impact on tumor biological behavior. The left side of the figure illustrates circRNA upregulation, where the proliferation and invasion & metastasis capabilities of tumor cells are significantly enhanced. In contrast, the right side depicts circRNA downregulation, leading to the suppression of tumor cell proliferation and invasion & metastasis. Overall, the differential expression levels of circRNAs regulate the malignant phenotypes of colorectal cancer cells, providing potential targets for subsequent diagnostic and therapeutic strategies.

CircRNAs exhibit robust and stable biological activities, playing diverse roles in physiological and pathological pathways. They can significantly influence the tumor microenvironment, affecting tumor growth and progression (22)(**Figure 1**). CircRNAs also impact the stem-like properties of cancer cells, contributing to cancer progression (23, 24). Dysregulated circRNA expression has been observed in various common and rare cancers (25, 26) (**Figure 1**). Exploring these alterations can deepen our understanding of circRNAs in cancer initiation and progression. Furthermore, specific tumor-associated circRNAs show promise as diagnostic biomarkers for cancer detection (27–29).

**Figure 2:**
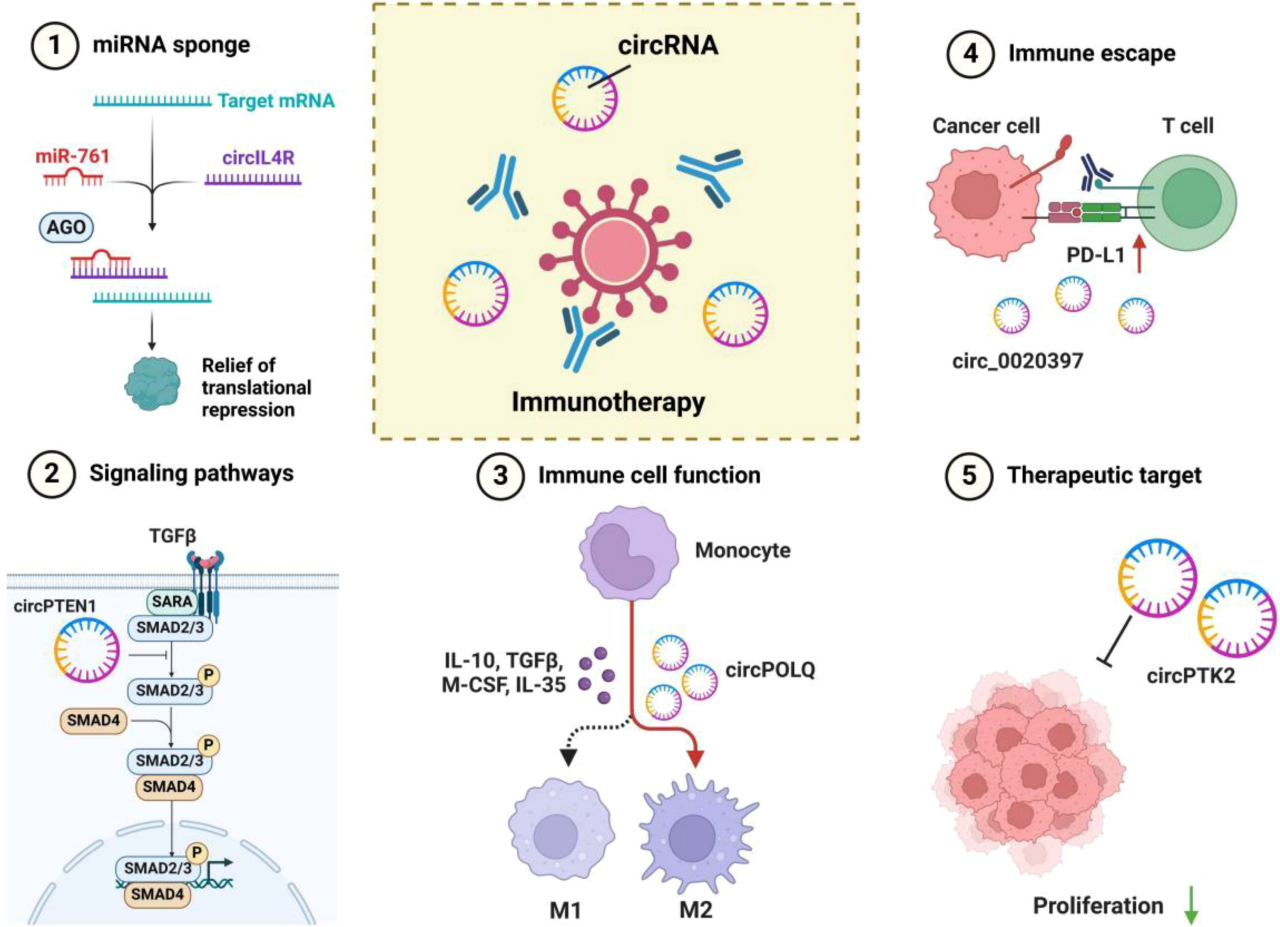
Multiple mechanisms of circRNA in tumor immunomodulation and immunotherapy. 1. miRNA Sponge: circRNAs (e.g., circIL4R) bind to specific miRNAs (e.g., miR-761), alleviating the translational repression of target mRNAs, thereby influencing tumor cell proliferation and invasion. 2. Signaling Pathway Regulation: circRNAs participate in regulating key signaling pathways (e.g., TGFβ/SMAD) by modulating the activity of transcription factors (e.g., SMAD2/3/4), further affecting cell proliferation, differentiation, and immune responses. 3. Immune Cell Polarization: Certain circRNAs (e.g., circPOLQ) can influence the polarization of macrophages into M1 or M2 phenotypes, shaping the immune landscape of the tumor microenvironment through the secretion of cytokines (e.g., IL-10, TGFβ, M-CSF, IL-35). 4. Immune Evasion: circRNAs (e.g., circ_0020397) interact with immune checkpoint pathways such as the PD-1/PD-L1 axis, promoting tumor cell evasion from immune surveillance and destruction. 5. Potential Therapeutic Targets: circRNAs (e.g., circPTK2) play a critical role in regulating tumor cell proliferation and immune responses, making them promising targets for novel strategies in tumor immunotherapy through modulation of their expression or function.

CircRNAs regulate gene expression through diverse mechanisms (30–32) (**Figure 2**): 1. miRNA Sponges: CircRNAs can act as competing endogenous RNAs (ceRNAs) by binding to miRNAs via enriched binding sites, modulating their activity. They may also serve as miRNA reservoirs or facilitate intracellular transport (10, 33). 2. Protein Sponges: Certain circRNAs bind RNA-binding proteins (RBPs), such as circMBL, which interacts specifically with MBL protein to regulate its own synthesis. This interaction depends on conserved binding sites within circMBL and its intronic sequences (19). 3. Protein Scaffolding: CircRNAs can bridge enzymes and substrates. For example, circFOXO3 binds both p53 and MDM2, enabling MDM2 to ubiquitinate p53 (34–36). 4. Template for Translation: CircRNAs with internal ribosome entry sites (IRES) can encode peptides. For instance, circPPP1R12A encodes a peptide that promotes colon cancer progression via Hippo-YAP signaling, while circFNDC3B-derived protein suppresses tumors by enhancing FBP1 activity (37–39). 5. Transcription Regulation: EIciRNAs, bound to U1snRNP, interact with RNA polymerase II to enhance transcription of their parental genes (40). 6. RNA Stability: circRNAs stabilize other RNAs, such as mRNAs and lncRNAs, often through protein interactions. For instance, circZNF609 recruits ELAV1 (HuR) to enhance mRNA stability and translation, while circXPO1 stabilizes CTNNB1 mRNA via IGF2BP recruitment, promoting lung adenocarcinoma progression (10, 41). 7. RBP Modulation: circRNAs can sequester RBPs in the cytoplasm, preventing their nuclear translocation or modulating their activity. In NSCLC, circNDUFB2 scaffolds TRIM25 and IGF2BPs to facilitate ubiquitination and degradation of IGF2BPs, activating anti-tumor immunity (42). Though most circRNAs are non-coding, some have translational potential under two conditions: the presence of open reading frames (ORFs) and IRES, or m6A modifications in their 5’UTR. Examples include circE-Cad, which encodes C-E-Cad to promote glioblastoma tumorigenicity via EGFR-STAT3 signaling, and circRNAs from oncogenic viruses like circE7, which translates the E7 oncoprotein in cervical and head and neck cancers (43–45).

In summary, an increasing body of research is delving into the biogenesis and functionality of circRNAs, with a particular focus on the cellular processes that give rise to circRNAs and the mechanisms by which they exert their influence on development and disease. These studies underscore the potential of circRNAs to serve as valuable diagnostic and prognostic biomarkers.

## 3 The interplay between circular RNAs and colorectal cancer

### 3.1 Variations in expression profiles

Research indicates that over 70 circRNAs are significantly upregulated in CRC, actively contributing to its initiation and progression. For example, circHERC4 is highly expressed in CRC tissues and correlates with lymph node metastasis and advanced tumors (46). CircHIPK3 is overexpressed in hepatocellular carcinoma, breast, CRC, and lung cancers, with elevated levels in CRC linked to poorer prognosis (47–49). Similarly, circALG1 is associated with CRC metastasis, while circPTK2 overexpression correlates with metastasis, advanced staging, and chemotherapy resistance (16, 50, 51). Increased circ5615 expression is linked to advanced T stage and poor prognosis in CRC (52). Conversely, downregulated circRNAs often exhibit anti-tumor effects in CRC. CircPTEN1 and circLPAR1 are both under expressed in CRC tissues, with the latter linked to reduced tumor weight and size (53, 54). CircPLCE1 downregulation is associated with poorer survival and advanced staging (45, 55, 56). CircEXOC6B, significantly downregulated in CRC, negatively correlates with tumor size, lymphatic metastasis, and TNM stage (57). CircLHFPL2 is also under expressed in PIK3CA-mutated CRC, with its downregulation linked to poor prognosis (58). Overall, the abnormal expression patterns of circRNAs in CRC highlight their potential as biomarkers for diagnosis and prognosis.

### 3.2 The mechanism of action

CircRNAs influence cancer development through various mechanisms shaped by their sequence, stability, post-transcriptional modifications, secondary structure, and accumulation patterns under specific conditions. They play critical roles in modulating tumor signaling pathways such as PI3K/AKT, Wnt/β-catenin, JAK/STAT, GEF-H1/RhoA, and TGF-β/Smad. CircRNAs regulate these pathways by upregulating oncogenes, downregulating tumor suppressor genes, or modulating downstream protein levels (49, 54, 59–63). As a result, circRNAs hold significant potential as biomarkers for CRC diagnosis.

This study reveals for the first time that circIL4R expression is significantly elevated in CRC cells, tissues, and serum, highlighting its potential as a diagnostic and prognostic biomarker. TFAP2C transcriptionally induces circIL4R expression, which competitively binds miR-761, thereby upregulating TRIM29. This process targets PHLPP1 for ubiquitin-mediated degradation, activating the PI3K/AKT pathway and promoting CRC progression (62). In contrast, circPTEN1 acts as a CRC suppressor by interfering with the TGF-β/Smad signaling pathway. CircPTEN1 binds to the MH2 domain of Smad4, disrupting its interaction with phosphorylated Smad2/3, thereby curbing metastasis driven by TGF-β signaling. Targeting TGF-β signaling could serve as an effective therapeutic strategy, with circPTEN1 emerging as a promising candidate for preventing metastatic CRC (52). Additionally, a positive feedback loop involving HIF1A, RRAGB, and mTORC1 plays a key role in CRC development. CircEXOC6B binds to RRAGB, disrupting its interaction with RRAGC/D and inhibiting this loop. This suppression hampers CRC cell growth and enhances 5-fluorouracil (5-FU)-induced apoptosis, offering new insights into therapeutic targets involving the HIF1A and mTORC1 pathways (64). The circular RNA circGPRC5A is significantly elevated in CRC tissues compared to normal counterparts and is strongly associated with tumor size, stage, and lymph node involvement. *In vitro* and *in vivo* studies revealed that circGPRC5A enhances CRC cell proliferation, migration, and metastasis. Mechanistically, circGPRC5A binds to PPP1CA, inhibiting its ubiquitination by UBA1 and preventing proteasomal degradation. This stabilizes PPP1CA, increasing its phosphatase activity, which dephosphorylates YAP at Ser127 and Ser109. Dephosphorylated YAP translocates to the nucleus, interacts with TEAD transcription factors, and activates target gene expression, promoting tumor progression. Immunohistochemical analysis further demonstrated elevated Ki-67 and PPP1CA expression in tumors with high circGPRC5A levels, consistent with its role in enhancing cell proliferation. Silencing circGPRC5A reduced these protein levels, confirming its involvement in CRC progression and tumorigenesis (65). A study confirmed the cyclic structure and intracellular localization of circFNDC3B, highlighting its reduced expression in colon cancer and its association with poorer patient survival. The research demonstrated that lower levels of circFNDC3B suppressed colon cancer progression. Analysis through circRNADb revealed that circFNDC3B contains ORFs and IRES, enabling it to encode proteins. Specifically, circFNDC3B-218aa was shown to inhibit metastasis and EMT in colorectal cancer by modulating the Snail/FBP1 signaling pathway, thereby suppressing tumor growth. Moreover, circFNDC3B-218aa promotes a metabolic shift from glycolysis to oxidative phosphorylation, further impeding EMT progression. These findings provide new insights into the mechanisms of colorectal cancer development (39).

Recent studies have revealed several critical roles of circRNAs in CRC. CircYAP, encoding the oncogenic protein YAP-220aa, promotes liver metastasis in CRC by inhibiting LATS1-mediated YAP phosphorylation, thereby enhancing YAP activity. Notably, circYAP is overexpressed in CRC with liver metastases and correlates with poor prognosis, positioning it as a potential prognostic biomarker and therapeutic target (66). CircHERC4, another oncogenic driver, is significantly upregulated in CRC tissues and linked to increased proliferation, migration, and invasiveness of CRC cells. Elevated circHERC4 levels are associated with metastasis and poor survival outcomes. Mechanistically, circHERC4 may inhibit miR-556-5p, thereby upregulating CTBP2 and suppressing E-cadherin activation. These findings suggest that targeting circHERC4 could offer novel therapeutic strategies (46). Conversely, circFBXW4 functions as a tumor suppressor in CRC by regulating the miR-338-5p/SLC5A7 axis, presenting a new avenue for therapy (67). Additionally, exosome-derived circLPAR1 suppresses CRC growth by binding to eIF3h, disrupting the METTL3-eIF3h interaction, and reducing BRD4 translation, providing new insights into early diagnosis and disease mechanisms (53). Finally, circFMN2 promotes CRC cell proliferation and migration through the miR-1182/hTERT pathway, highlighting the intricate regulatory network between circRNAs, miRNAs, and mRNAs in CRC progression (68).

In aggregate, these findings indicate that individual circRNAs could exert both pro- and anti-cancer effects in the context of colorectal carcinogenesis, metastasis, and drug resistance, mediated by diverse molecular pathways. Consequently, they may represent promising and valuable biomarkers for the clinical diagnosis, treatment, and prognostic evaluation of cancer.

## 4 The potential of circRNA in immunotherapy for CRC

### 4.1 Immunomodulation

#### 4.1.1 The role of circRNAs in modulating immune responses within the tumor microenvironment

CircRNAs play a crucial role in modulating immune responses within the tumor microenvironment (TME) (69). Studies show that various immune cells, including monocytes, neutrophils, B-cells, and platelets, can internalize extracellular circRNAs, with monocytes exhibiting the highest uptake efficiency. The uptake of circRNAs is particularly enhanced in differentiated macrophages and dendritic cells (DCs), indicating their key role in recognizing and processing circRNAs (70). CircRNAs positively regulate macrophage function and, when derived from tumor cells, can induce M2 polarization in macrophages (71). This polarization is governed by pathways such as JAK1/STAT3 and PI3K-AKT, with circRNAs actively participating in these processes (72, 73). M2 macrophages, in response to tumor-associated cytokines, lose their antitumor function and instead secrete immunosuppressive factors like IL-10, TGF-β, and IDO, promoting immune evasion (74). Additionally, exosomal circRNAs from macrophages, such as circMERTK, enhance IL-10 production in tumor-associated macrophages (TAMs), which suppresses CD8+ T-cell function and contributes to the immunosuppressive TME. Targeting this mechanism could offer therapeutic potential in colorectal cancer (75).

**Figure 3:**
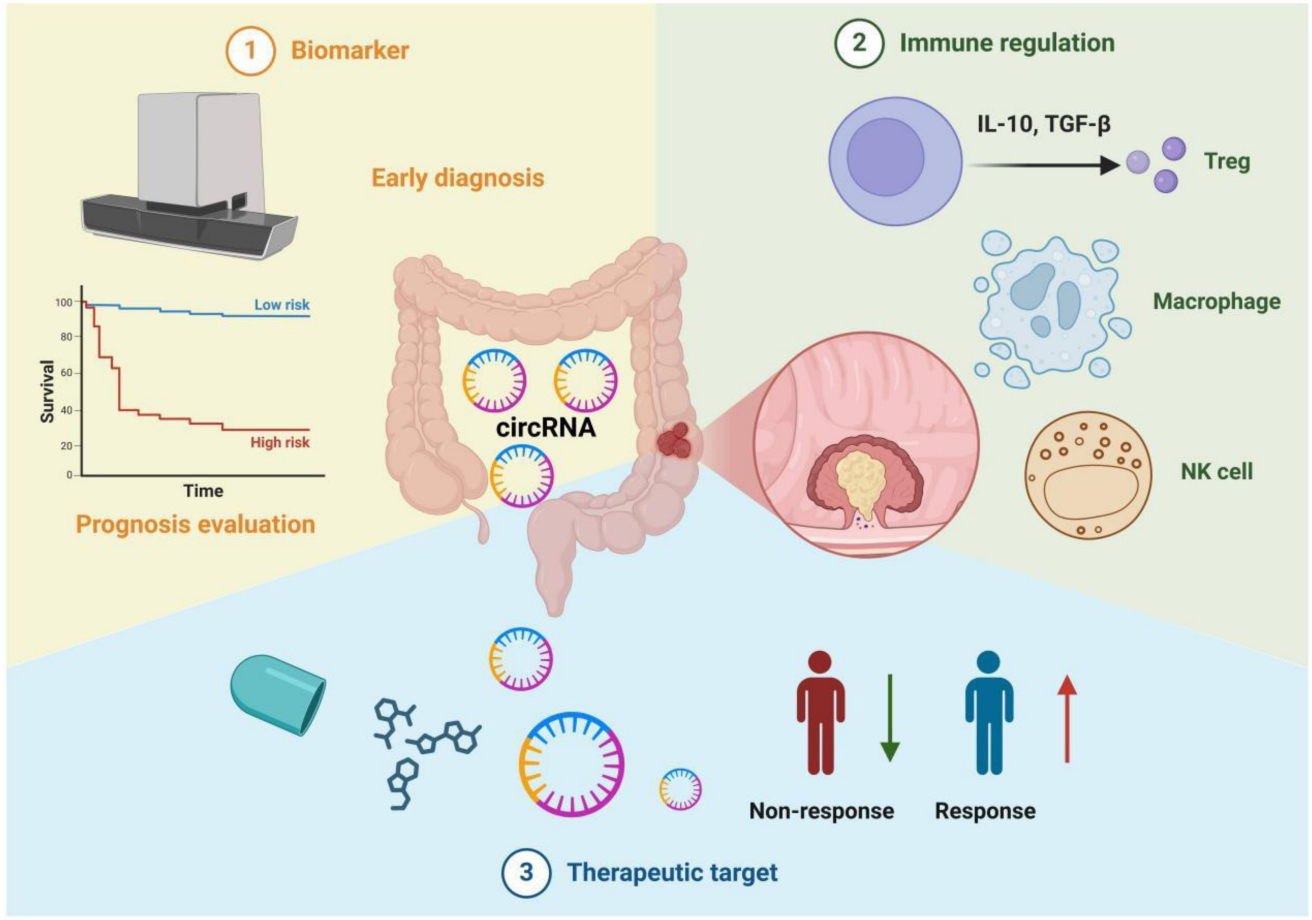
Multifunctionality of circRNAs in colorectal cancer immunotherapy. 1. Biomarkers: circRNAs can be utilized for early diagnosis and prognosis evaluation. For instance, by detecting circRNA expression levels in blood or tissue samples and correlating them with clinical survival curves, patients can be stratified into high-risk and low-risk groups. 2. Immune Regulation: circRNAs influence the balance of the tumor immune microenvironment by modulating the functions of various immune cells, including regulatory T cells (Tregs), macrophages, and natural killer cells (NK cells). They also promote or suppress tumor progression by inducing or inhibiting the secretion of cytokines (e.g., IL-10, TGF-β). 3. Therapeutic Targets: Molecular interventions targeting circRNAs hold promise for improving tumor treatment efficacy. The figure contrasts "Non-response" and "Response," illustrating that some patients achieve better therapeutic outcomes under circRNA-targeted strategies. This highlights the potential of circRNAs as emerging targets for precision therapy in colorectal cancer.

CircRNAs play a key role in enhancing tumor immunity by promoting T cell recruitment and activation (**Figure 3**). For example, circDNA2v, frequently overexpressed in CRC, prevents its own ubiquitination and degradation by binding to IGF2BP3, stabilizing c-Myc mRNA and influencing the oncogenic traits of CRC cells. Knockdown of circDNA2v activates the JAK-STAT1 pathway and increases the secretion of CXCL10 and IL-9, which enhance the chemotactic and cytotoxic functions of CD8+ T cells, boosting anti-tumor immunity, as shown in *in vitro* and *in vivo* models (76). On the other hand, certain circRNAs, such as circRNF216, can inhibit CRC progression and enhance tumor immunity. circRNF216 promotes CD8+ T cell infiltration by upregulating ZC3H12C, triggering an immune response that helps limit tumor growth (77). Thus, circRNAs may serve as potential biomarkers for CRC treatment by modulating tumor immunity.

A study found that transfection of circARGL into CRC cells enhances their proliferation and migration. Exosomal circARGL also affects TGF-β expression, suggesting its role in tumor progression (78, 79). TGF-β, a multifunctional cytokine, regulates immune functions and is linked to tumorigenesis and metastasis. In the tumor microenvironment, TGF-β suppresses N1 neutrophil differentiation while promoting N2 neutrophils, aiding tumor progression (80, 81). Exosomal circRNAs from tumors modulate TGF-β expression through miRNA sponging, contributing to the N1 to N2 switch and supporting tumor growth (82).

Natural Killer (NK) cells, crucial to immune defense, are impacted by exosomal circRNAs such as circFOXO3 and circRHOT1, which sequester miRNAs, leading to NK cell senescence and tumor progression (83). Exosomes from cancer cells, carrying circUHRF1, impair NK cell function and contribute to resistance to anti-PD1 therapy in hepatocellular carcinoma. Additionally, elevated circFAT1 expression activates STAT3, reducing CD8+ T cell infiltration and diminishing PD1-blocking immunotherapy efficacy, promoting immune evasion (84). Targeting circRNAs and modulating immune cells in the tumor microenvironment could enhance immunotherapy efficacy and improve patient survival. In summary, circRNAs play a central role in regulating tumor-associated macrophages, regulatory T cells, CD8+ T cells, neutrophils, and NK cells in the tumor microenvironment.

#### 4.1.2 The role of circular RNAs in modulating immune responses and facilitating or impeding immune evasion

The TME enables tumor cells to infiltrate blood and lymphatic vessels, evading immune surveillance and resisting T cell cytotoxicity, leading to metastasis and tumor growth (64). In CRC, circ_0020397 sponges miR-138, upregulating TERT and PD-L1 expression (85). CiR7 increases PD-L1 levels through miRNA-independent modulation of CMTM4 and CMTM6 (64), while hsa_circ_0136666 and circ-KRT6C enhance PD-L1 expression by targeting miR-497 and miR-485-3p, respectively (86, 87). Elevated TERT expression promotes CRC cell proliferation, while PD-L1 suppresses T cell activation, aiding immune evasion and cancer progression (88). Silencing circPGPEP1 boosts T cell proliferation and inhibits CRC tumor growth (64). Overexpression of circQSOX1 promotes glycolysis and reduces the effectiveness of anti-CTLA-4 therapy, aiding immune escape (89). These findings suggest that circRNAs could serve as promising diagnostic biomarkers for CRC.

### 4.2 Therapeutic

#### 4.2.1 Therapeutic targeting of circRNAs and immunotherapeutic strategies

CircRNAs have emerged as critical regulators in colorectal cancer (CRC), serving as both therapeutic targets and immunomodulators. Oncogenic circRNAs, such as CDR1as and circHIPK3, promote CRC progression by sponging tumor-suppressive miRNAs (e.g., miR-7) and activating pathways like EGFR/IGF1R and FAK/YY1 (90, 91). Silencing these circRNAs using siRNAs or shRNAs (e.g., against circPTK2 and circMETTL3) effectively suppresses tumor growth and metastasis (15, 92–94). Additionally, circRNAs like circKRT6C and circQSOX1 modulate immune evasion mechanisms, such as the miR-485-3p/PD-L1 axis and Treg-mediated immunosuppression, highlighting their potential to enhance immunotherapy (87, 89). Tumor-suppressive circRNAs, including circDDX17 and circ-FBXW7, inhibit CRC proliferation when overexpressed, offering alternative therapeutic strategies (90).

CircRNA-based immunotherapies are showing promising preliminary outcomes (**Table 1**). For instance, combining PD-1/PD-L1 blockade with CDR1as targeting enhances immunotherapy efficacy by upregulating PD-L1 expression (15, 92). Small molecules YAP inhibitors suppress circPPP1R12A-73aa impairing tumor metastasis (95). Exosome-derived circRNAs and radiation-induced circRNA modulation (e.g., carbon ion irradiation) provide novel diagnostic and therapeutic biomarkers (96, 97). Furthermore, CRISPR-based editing (e.g., circZNF800 knockdown) and circRNA cloning into plasmid vectors demonstrate potential in CRC treatment (17, 92).

**Table 1:** CircRNA-mediated immunotherapy in CRC.

circRNAs	Immunotherapy relevance	Main biological pathways/mechanisms	References
circRNA_0020397	Inhibition of PD-L1 expression and enhancement of T cell activity by miR-138	miR-138/PD-L1 signaling axis	(85)
circIL4R	Potential enhancement of T cell or NK cell function by adsorption of miR-761, etc., attenuating inhibition of immune-related genes	miRNA sponge mechanism (regulation of immune effector gene expression)	(121)
circPOLQ	Modulation of macrophage M1/M2 polarization affects the balance between immunosuppression and pro-inflammatory responses in the tumor microenvironment	Inflammatory cytokine secretion (IL-10, TGF-β, etc.)	(74)
circPTK2	Involved in the proliferation and metastasis of colon cancer cells and may have a key regulatory role in immune cell infiltration and therapeutic response	PTK2 signaling pathway (adhesion, migration and immune cell recruitment)	(94)
circBtnl1	Negative regulation of intestinal stem cell self-renewal may indirectly regulate the tumor microenvironment by affecting stem cell stemness	Binding Atf4 mRNA inhibits its stability, downregulates Sox9 expression, and suppresses stem cell proliferation	(122)

These advancements underscore the dual role of circRNAs as therapeutic targets and immunomodulators. By targeting oncogenic circRNAs and leveraging tumor-suppressive circRNAs, researchers are developing innovative strategies to improve CRC outcomes. The integration of circRNA-based therapies with existing treatments, such as immune checkpoint inhibitors and small molecules, holds significant promise for advancing CRC immunotherapy and reducing disease burden (64, 86, 88, 89, 93, 98–101).

### 4.3 Prognostic evaluation

#### 4.3.1 The potential of circular RNA as a prognostic biomarker in immunotherapy for CRC

CircRNAs are emerging as promising biomarkers for cancer prognosis due to their high abundance and stability in cancer cells, solid tumors, and body fluids, including serum, plasma, and urine (15). Certain circRNAs correlate with clinicopathological features like lymphatic metastasis, distant metastasis, and recurrence, making them potential prognostic biomarkers for CRC (92). For example, upregulation of circ3823 is linked to enhanced proliferation, metastasis, and angiogenesis, while circ5615 correlates with T-staging (15). CircHIPK3, when upregulated, boosts CRC cell proliferation, migration, invasion, and apoptosis. circSPARC’s overexpression associates with larger tumors, deeper infiltration, and poor survival (15). Conversely, downregulation of circPTEN1 promotes metastasis and invasion, serving as an independent predictor of poor survival outcomes (102). Other circRNAs, including circ_0009361, can suppress CRC growth and metastasis, indicating their potential as prognostic biomarkers (15). CircRNAs like circCCDC66, circPPP1R12A, ciRS-7, and circ_0014717 correlate with reduced survival rates, emphasizing their prognostic value in CRC (90). A circular RNA-based classifier (cirScore) using four circRNAs (hsa_circ_0122319, hsa_circ_0087391, hsa_circ_0079480, hsa_circ_0008039) has been developed to predict CRC recurrence (92, 103). Additionally, hsa_circ_0005075 and circFADS2 serve as independent predictors of CRC prognosis (104, 105). CircHIPK3 and circCCDC66 are particularly promising, with roles in CRC cell growth and metastasis, and their expression is inversely related to clinical outcomes (106). Finally, hsa_circRNA_102958 promotes CRC cell proliferation and invasion, suggesting it may also be a biomarker for poor prognosis. Overall, circRNAs offer exciting potential for prognostic assessment and therapeutic targeting in CRC (107).

#### 4.3.2 The role of circRNAs in predicting treatment efficacy and disease progression

Numerous downregulated circRNAs play a critical role in negatively regulating CRC growth and metastasis. Due to their stability and long half-life, these tumor suppressor circRNAs could have substantial antitumor effects when expressed in CRC cells or tissues. Zheng et al. found that circLPAR1 expression was significantly reduced in CRC tissues, and its overexpression decreased tumor weight and size, suggesting its potential as a biomarker for poor prognosis (15). Similarly, circRNF216, downregulated in CRC, inhibits metastasis when overexpressed, both *in vitro* and *in vivo (* 77). CircRERE suppresses CRC malignancy by sequestering miR-6837-3p, upregulating MAVS, and enhancing the type I IFN signaling pathway, thus stimulating anti-tumor immunity. This effect is further potentiated when combined with anti-PD-1 therapy (18). Additionally, hsa_circRNA _00004677, upregulated in CRC tissues, contributes to tumor progression by promoting eIF4A3-driven translation of the c-Myc oncogene, correlating with poor patient prognosis (108). In conclusion, circRNAs are crucial modulators of tumorigenesis in various cancers. Their stability and tissue-specific expression highlight their potential as molecular biomarkers and therapeutic targets, meriting further investigation for early diagnosis, treatment, and prognostic assessment in CRC (89).

## 5 Bioinformatics tools in circRNA research

### 5.1 Integrating bioinformatics tools in circRNA research

The integration of bioinformatics tools has significantly advanced the study of circRNAs in CRC, providing robust platforms to analyze circRNA-miRNA-mRNA networks and regulatory interactions. Tools such as CircNet 2.0 and CircNetVis enable comprehensive visualization and reconstruction of circRNA-centered regulatory networks, identifying key sponging interactions and downstream mRNA targets (109, 110). CircScan and EasyCircR facilitate accurate circRNA identification and quantification from RNA-seq data, distinguishing circRNAs from linear isoforms with high precision (111). Specialized tools like circRNA-sponging and CRAFT predict miRNA-binding sites and functional enrichment, aiding in prioritizing circRNAs with high sponging potential or disease relevance (112). Additionally, circMine and riboCIRC offer user-friendly interfaces for exploring circRNA expression profiles, clinical correlations, and cross-species conservation, streamlining hypothesis generation (113, 114). These tools collectively enhance the systematic exploration of circRNA roles in CRC pathogenesis.

### 5.2 Complementing experimental findings

Bioinformatics tools bridge the gap between high-throughput data and mechanistic insights, complementing wet-lab experiments. For example, CircNet 2.0 can validate experimentally observed circRNA-miRNA interactions by mapping them to established networks, reinforcing their biological significance (91). CRAFT and circRNA-sponging predict novel interactions that guide targeted functional studies, reducing trial-and-error approaches (87). Tools like riboCIRC integrate ribo-seq data to assess circRNA translatability, supporting findings on oncogenic circRNA-encoded peptides (95). Furthermore, circMine links circRNA expression with patient survival data, helping prioritize biomarkers for clinical validation (93, 94). By contextualizing experimental results within larger regulatory frameworks, these tools enhance the reproducibility and translational relevance of circRNA studies.

### 5.3 Accelerating circRNA research and clinical translation

The synergy between bioinformatics tools and experimental research accelerates the discovery of circRNA-based diagnostics and therapies. EasyCircR and CircScan standardize circRNA detection pipelines, enabling consistent analysis across studies and cohorts (111). CircNetVis generates interactive networks to visualize circRNA-driven immune evasion mechanisms, aiding in the design of combination therapies targeting circRNAs and immune checkpoints (110). Databases like circMine provide pre-processed multi-omics datasets, allowing researchers to explore circRNA-drug interactions or repurpose existing therapies (115). Moreover, riboCIRC’s integration of translatome data supports the development of circRNA-encoded peptide-targeted therapies (114). By offering scalable, data-driven insights, these tools reduce research bottlenecks and foster innovation in CRC circRNA biology, ultimately accelerating the translation of circRNA discoveries into clinical applications.

## 6 Conclusions and prospects

**Figure 4:**
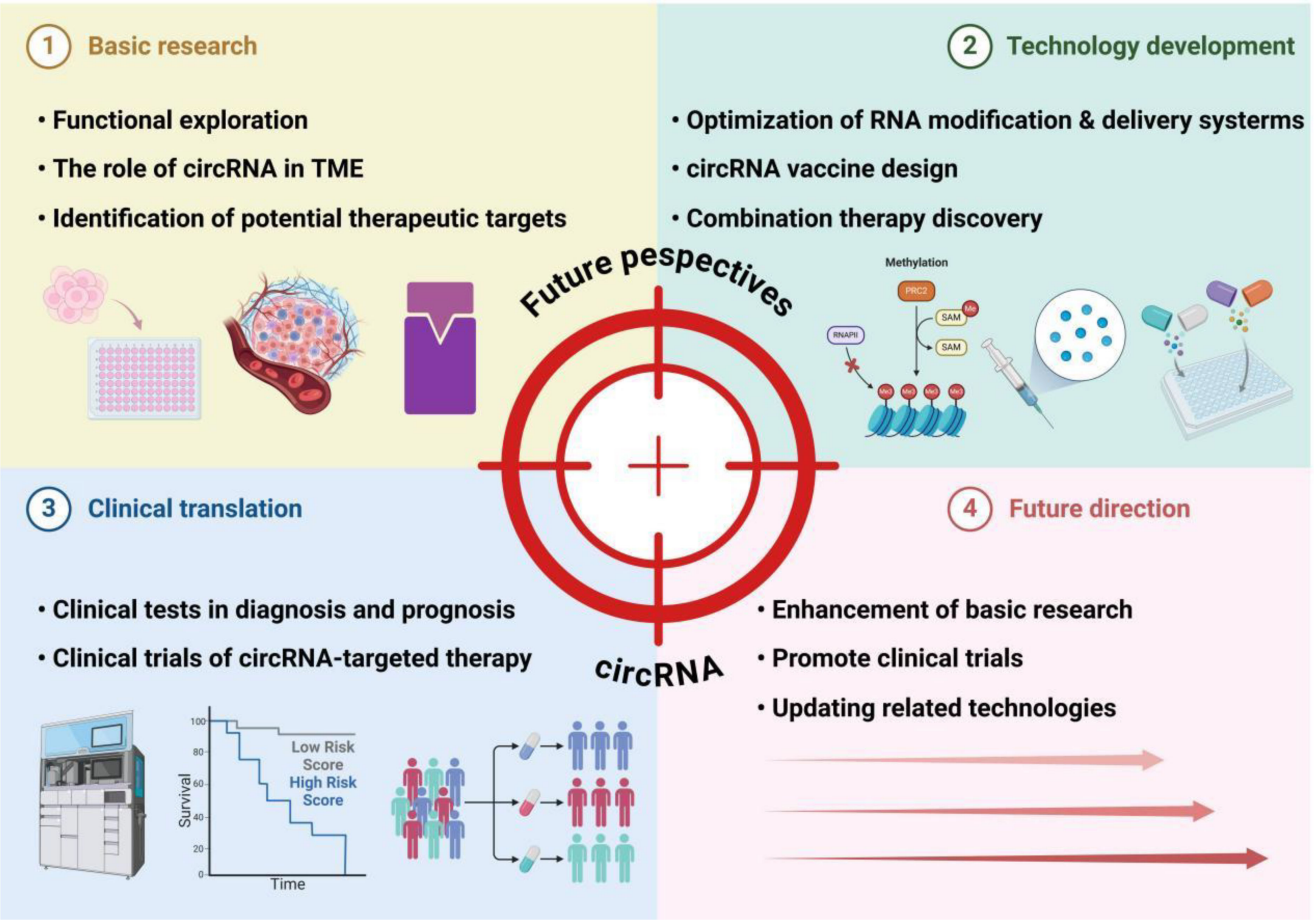
Future perspectives of circRNA in colorectal cancer immunotherapy. This framework outlines the multifaceted landscape of circRNA research and its applications. Basic research focuses on functional exploration, particularly the roles of circRNAs in the tumor microenvironment (TME) and the identification of therapeutic targets. Technology development encompasses optimizing RNA modification and delivery systems to enhance circRNA stability, designing circRNA-based vaccines, and discovering combination therapies to amplify treatment efficacy. Clinical translation advances circRNAs into practical use through biomarker-driven diagnostic and prognostic tests, as well as clinical trials evaluating the safety and efficacy of circRNA-targeted therapies. Looking forward, future directions emphasize deepening mechanistic understanding through enhanced basic research, expanding clinical trials to validate therapeutic potential, and updating technologies to refine circRNA applications. Collectively, these efforts bridge fundamental discoveries to clinical innovations, positioning circRNAs as pivotal tools in advancing cancer diagnosis and precision therapy.

The development of CRC is a multifactorial process driven by genetic, environmental, dietary factors, and dysregulated gene expression (**Figure 4**). As a novel class of non-coding RNAs, circRNAs exert pleiotropic effects in the CRC tumor microenvironment by regulating cell signaling, epithelial-mesenchymal transition, angiogenesis, and immune evasion, demonstrating potential as diagnostic/prognostic biomarkers (88, 106). Although research on circulating RNAs in CRC remains nascent, circRNAs have shown unique therapeutic value: 1) acting as competitive inhibitors of microRNAs to modulate immune responses (77); 2) serving as stable vaccine vectors encoding tumor antigens to enhance anti-tumor immunity via activation of CD8+/CD4+ T cells and dendritic cells (116–118); and 3) synergizing with combination therapies such as CAR-T (chimeric antigen receptor T-cell) (119, 120).

Current limitations and challenges include:

1. Predominant reliance on *in vitro* models for mechanistic studies and a lack of clinical-grade delivery systems;
2. Constraints in validating *in vivo* effects due to interspecies microenvironmental disparities;
3. Clinical translation risks.

Proposed strategies for advancement:

1. Development of novel vectors to enable sustained circRNA expression;
2. Integration of single-cell sequencing and spatial omics to dissect spatiotemporal regulatory networks;
3. Establishment of standardized protocols for circRNA synthesis, purification, and delivery.

Although current studies have preliminarily revealed the potential of circRNAs in regulating immune responses in CRC, the hierarchy of evidence and clinical applicability still require cautious evaluation. Most mechanistic investigations rely on *in vitro* cell models, which fail to recapitulate the dynamic interactions between immune cells and stromal components in the tumor microenvironment. While animal experiments partially validate *in vivo* effects, interspecies differences in immune microenvironments may compromise the clinical extrapolation of findings. Furthermore, clinical data directly linking circRNAs to immunotherapy responses in CRC remain scarce. Some mechanisms are extrapolated from other cancer types, and existing clinical studies are predominantly limited by retrospective designs and small sample sizes, rendering them prone to confounding factors. Future research should integrate single-cell sequencing, spatial transcriptomics, and prospective cohorts to systematically dissect the spatiotemporal-specific roles of circRNAs in the human CRC immune microenvironment. Additionally, establishing standardized circRNA detection and functional validation protocols will be critical to enhance the translational value of these findings.
